# Identification of new fisetin analogs as kinase inhibitors: Data on synthesis and anti-skin cancer activities evaluation

**DOI:** 10.1016/j.dib.2021.106858

**Published:** 2021-02-10

**Authors:** Tithi Roy, Samuel T. Boateng, Sergette Banang-Mbeumi, Pankaj K. Singh, Pratik Basnet, Roxane-Cherille N. Chamcheu, Federico Ladu, Isabel Chauvin, Vladimir S. Spiegelman, Ronald A. Hill, Konstantin G. Kousoulas, Bolni Marius Nagalo, Anthony L. Walker, Jean Fotie, Siva Murru, Mario Sechi, Jean Christopher Chamcheu

**Affiliations:** a School of Basic Pharmaceutical and Toxicological Sciences, College of Pharmacy, University of Louisiana - Monroe, Monroe, LA 71209-0497, USA; b School of Clinical Sciences, College of Pharmacy, University of Louisiana at Monroe, Monroe, LA 71209-0497, USA; c Chemistry, School of Sciences, University of Louisiana at Monroe, Monroe, LA 71209-0497, USA; d Department of Chemistry and Pharmacy, University of Sassari, Via Vienna 2, 07100 Sassari, Italy; e Department of Pediatrics, Division of Pediatric Hematology/Oncology, Pennsylvania State University College of Medicine, Milton S. Hershey Medical Center, Hershey, Pennsylvania 17033-0850, USA; f Division of Biotechnology and Molecular Medicine, School of Veterinary Medicine, Louisiana State University, Baton Rouge, LA 70803, USA; g Department of Pathobiological Sciences, School of Veterinary Medicine, Louisiana State University, Baton Rouge, LA 70803, USA; h Division of Hematology and Medical Oncology, Mayo Clinic Hospital, Phoenix, AZ, 85054, USA; i Department of Chemistry and Physics, Southeastern Louisiana University, Hammond, LA 70402-0878, USA

**Keywords:** Anticancer evaluation, skin cancers, fisetin analogs, target prediction, computational docking, kinase activities, melanoma and epidermoid carcinoma

## Abstract

This article contains supplemental datasets of the recently published related research article *“Synthesis, Inverse Docking-Assisted Identification and in vitro Biological Characterization of Flavonol-based Analogs of Fisetin as c-Kit, CDK2 and mTOR Inhibitors against Melanoma and Non-melanoma Skin Cancers”* by Roy et al., [1]. It provides in-depth data not included in the original co-submission on the biophysical, molecular docking, and biological characterization of newly synthesized flavonol-based analogs of fisetin, a natural dietary small molecule with anticancer and anti-inflammatory properties. These synthetic small molecules were investigated as new, potential single and/or multi-kinase inhibitors of the cyclin-dependent kinase-2 (CDK2), receptor tyrosine kinases (c-KITs), and mammalian targets of rapamycin (mTOR) targets, potentially active against melanoma or non-melanoma skin cancers. Furthermore, this data-in-brief article comprises additional sets of results on several aspects of the properties of the dual and multiple kinase inhibitor compounds’ effects that were not presented in the associated article, including the activated targets that are dysregulated in skin cancers; the effects on markers of apoptosis; on colony formation; and in scratch wound healing assays. The study has identified a panel of novel fisetin analogs that are either single- or multi-kinase inhibitors, which may be further developed as active for the treatment of melanoma and non-melanoma skin cancers. The dataset presented herein will be utilized for additional studies aiming to establish a biological platform to steer for predictive and experimental screening of novel flavonoids and analogs in relevant organoids, humanized animal models and *in vivo* disease models. The present results should also serve as a key stepping-stone towards enabling target-structure-based design, synthesis and initial testing of novel analogs or derivatives of fisetin. The current study may eventually lead to the development of safe, promising and preclinical candidate entities for treatment of skin and other forms of cancers as well as various other human diseases, which can possibly add to the general armamentarium of promising and safe drugs for health promotion.

## Specifications Table

**Table:** 

Subject	Chemistry and Biological sciences
Specific subject area	Medicinal Chemistry, Molecular Biology and Skin Cancer Research
Type of data	Table Figure
How data were acquired	Dataset was collected as part of a synthesis and biophysical and biological characterization of novel flavonol derivatives of fisetin targeting specific kinases as anticancer agents [1]. The synthesized flavonol derivatives produced from microwave-assisted methods were purified and characterized using different spectroscopic and spectrometric techniques, such as ^1^H-NMR, ^13^C-NMR, IR, GC-MS and HR-ESIMS [1]. Cell cultures were prepared, analyzed, and protein content was quantified using the BCA reagent assay (Thermo Fisher Scientific, Waltham, MA, USA). Protein electrophoresis: SDS-PAGE method (Protean 3 system, Bio-Rad, Hercules, CA, USA), followed by protein expression via the western blotting method, using the Bio-Rad analysis and imaging system as earlier described [1,2]. Analysis and graphs in the figures, including *in vitro* enzymatic kinase activities, western blot, scratch
	wound assays, and colony formation were plotted using GraphPad PRISM program suite, version 8 (GraphPad Software, Inc., La Jolla, CA, USA), and molecular docking analysis [1,2]. *In silico* prediction of skin permeation, lipophilicity, absorption, distribution, metabolism, excretion (ADMET) properties of the analogs compared to the reference drug fisetin were performed and analyzed using the online SwissADME platform of the Swiss Institute of Bioinformatics [3].
Data format	Raw Analyzed
Parameters for data collection	Data were collected at the end of each experiment; each experiment was repeated at least three times. The post experiment analysis parameters for consideration were: effect or no effect, with statistical consideration for any significant differences.
Description of data collection	For western blot data, when exposed, faint and auto-exposed bands were obtained; the images were captured and analyzed using the Biorad image analysis software system. Densitometric data were collected, tallied and graphs were plotted to compare changes in the different treatment groups. The variations in protein and enzymes expressions in lysates and the differences in the proportion of colony sizes and numbers and wound closure areas were analyzed as in the associated research article [1] and as reported earlier [2]. Differences between samples treated with and without test compounds were measured and analyzed including protein and enzyme expression kinetics.
Data source location	Institution: University of Louisiana-Monroe City/Town/Region: Monroe, Louisiana Country: USA
Data accessibility	With the article
Related research article	T Roy, ST. Boateng, SBanang-Mbeumi, PK. Singh, P Basnet, RN Chamcheu, F Ladu, I Chauvin, VS. Spiegelman, RA. Hill, KG Kousoulas, BM Nagalo, AL Walker, J Fotie, S Murru, M Sechi, JC Chamcheu. Synthesis, inverse-docking assisted targets identification, and in vitro biological characterization of potent fisetin analogs as c-Kit, CDK2 and mTOR inhibitors active against melanoma and non-melanoma skin cancers. Bioorg Chem. 2020 Dec 30;107:104595. https://doi.org/10.1016/j.bioorg.2020.104595.

## Value of the Data

• This additional dataset provides further insights into the characteristics of the different flavonol compounds synthesized as single or multi-kinase inhibitors. These compounds exhibited varying degrees of biophysical properties and biological effects on human skin cancer cell lines compared to minimal effects on untreated control groups. The data provide a clear picture of the range diversity of the effects of the different synthetic small molecular structures examined.
• This data-in-brief article contains results that can be used by scientists and researchers as a reference and guide towards future investigations that may explore the potential therapeutic diversity as well as repurposing of these compounds for treating other human diseases.
• This dataset may help to formulate new additional research hypotheses to explore the pre-clinical effects of these compounds toward ultimate clinical use. Also, scientists can utilize the data to improve drug discovery and development in anticancer and adjuvant therapies, alone or in combination with other known drugs.
• The work presented informs further investigations on the toxicological and safety profiles in lieu of their formulations alone or in combination with existing FDA approved drugs.

## 1 Data Description

**Fig. 1:**
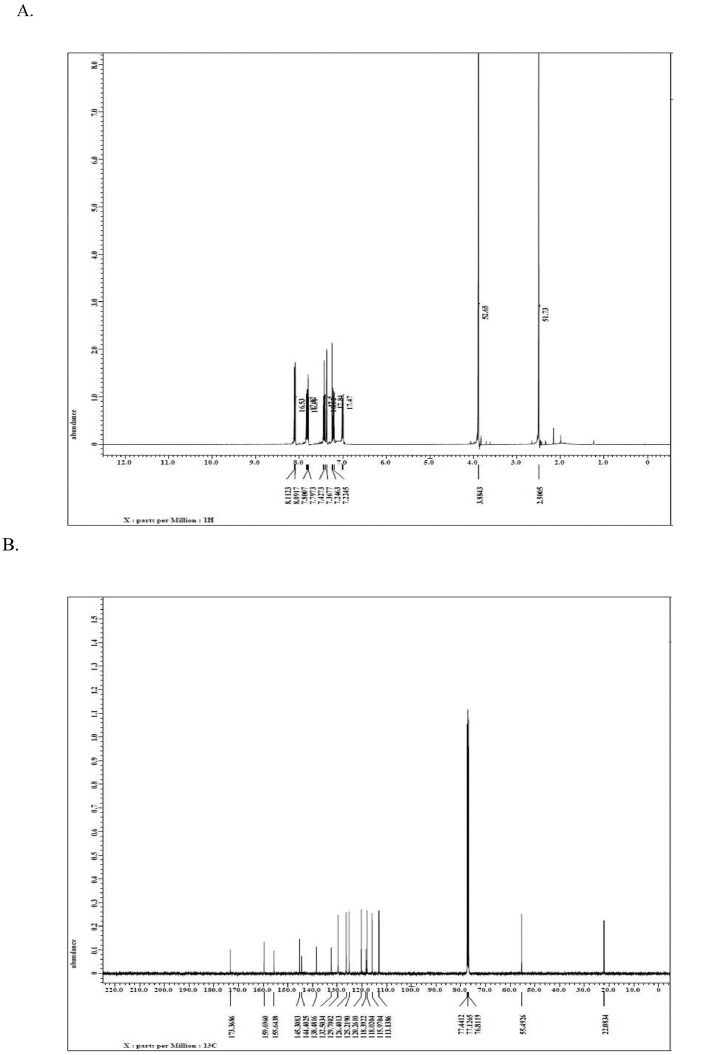

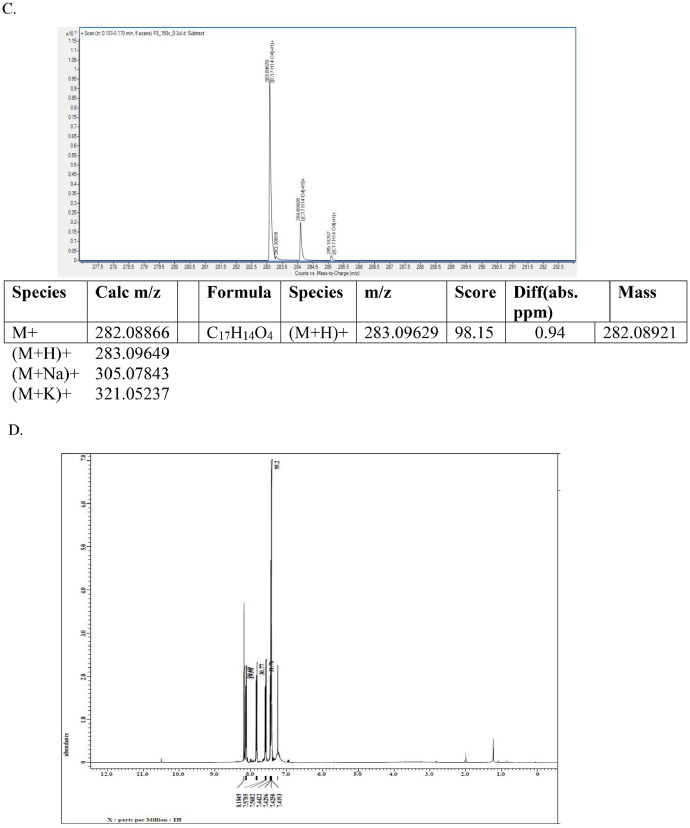

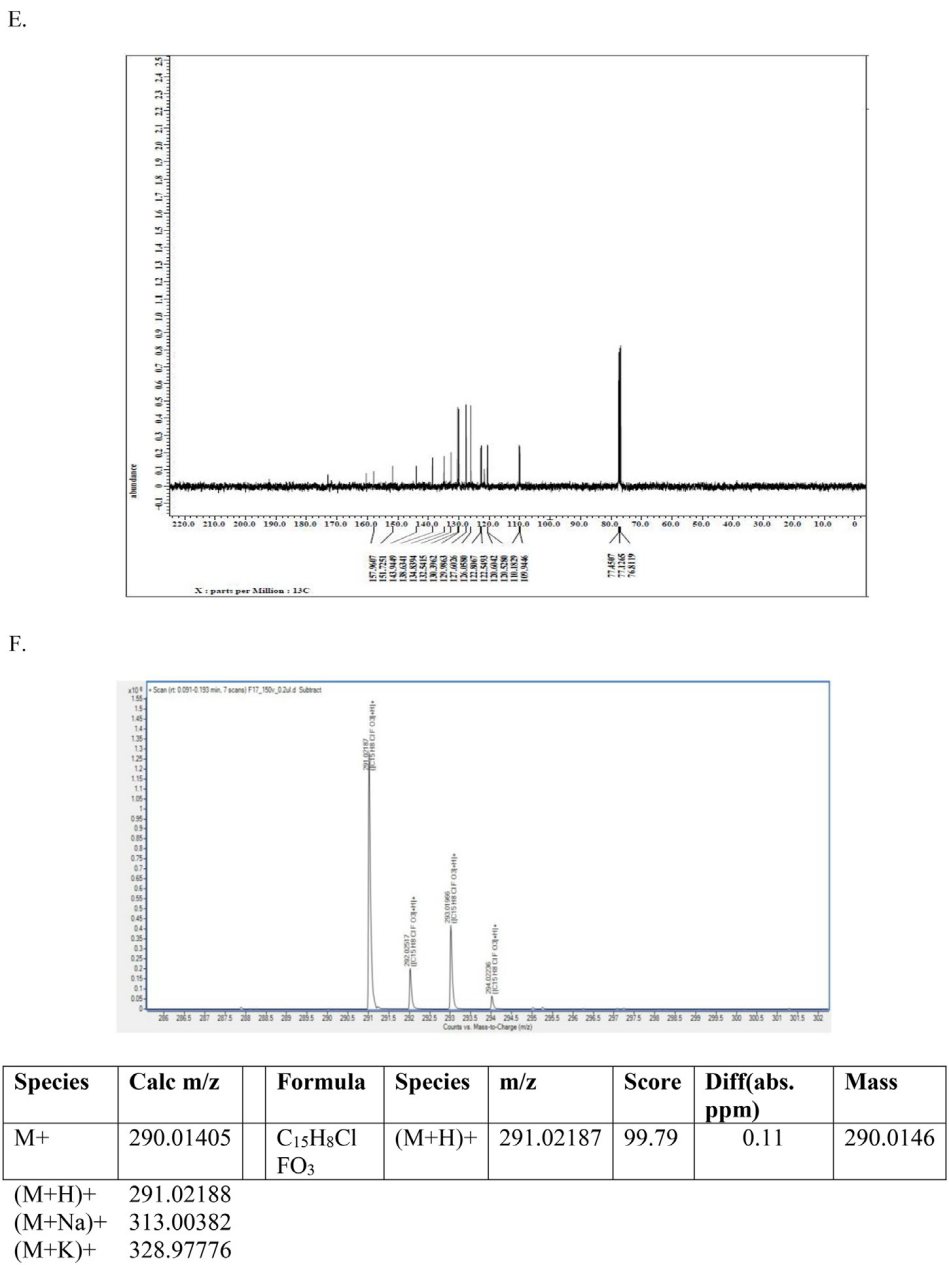

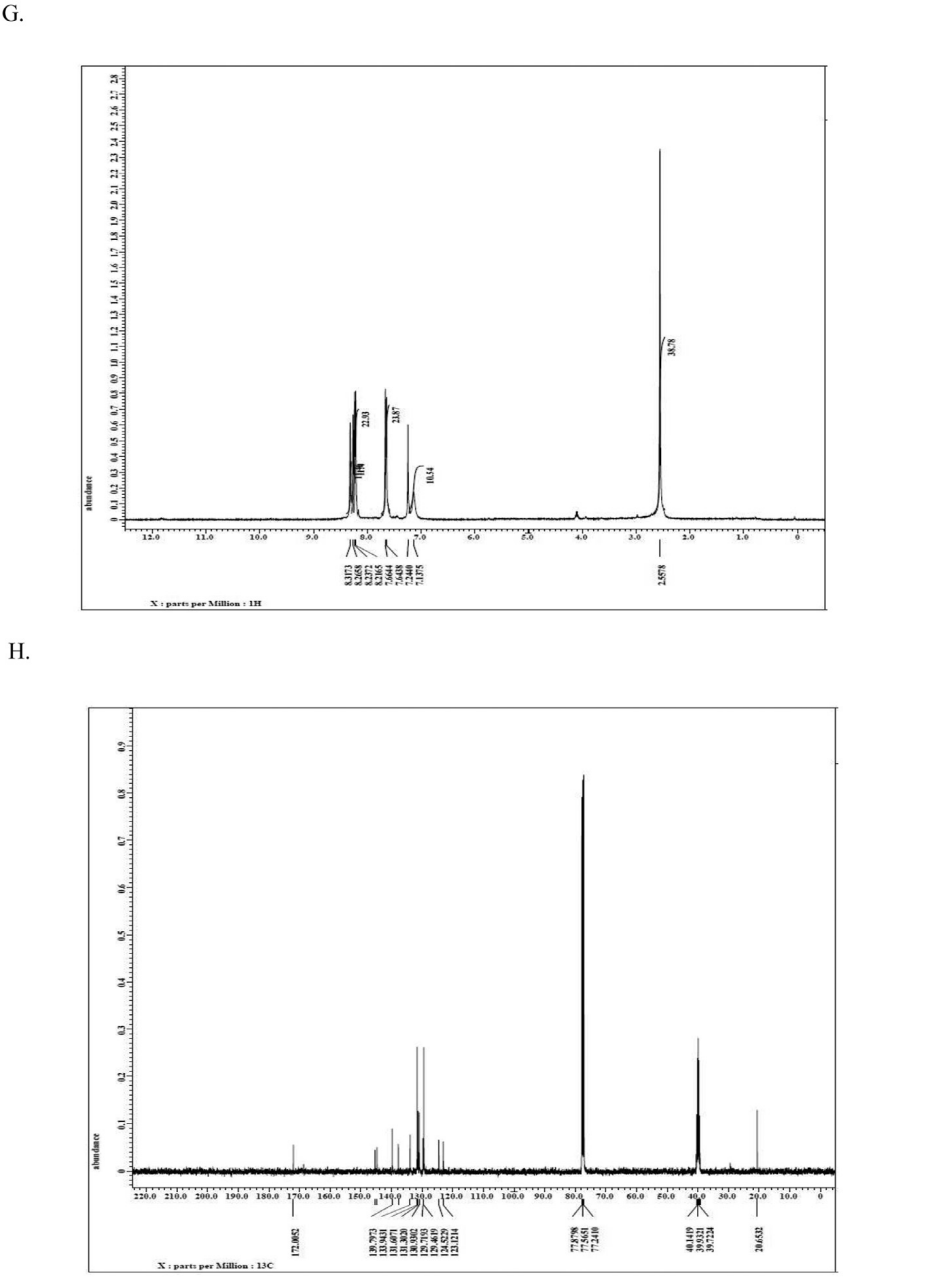

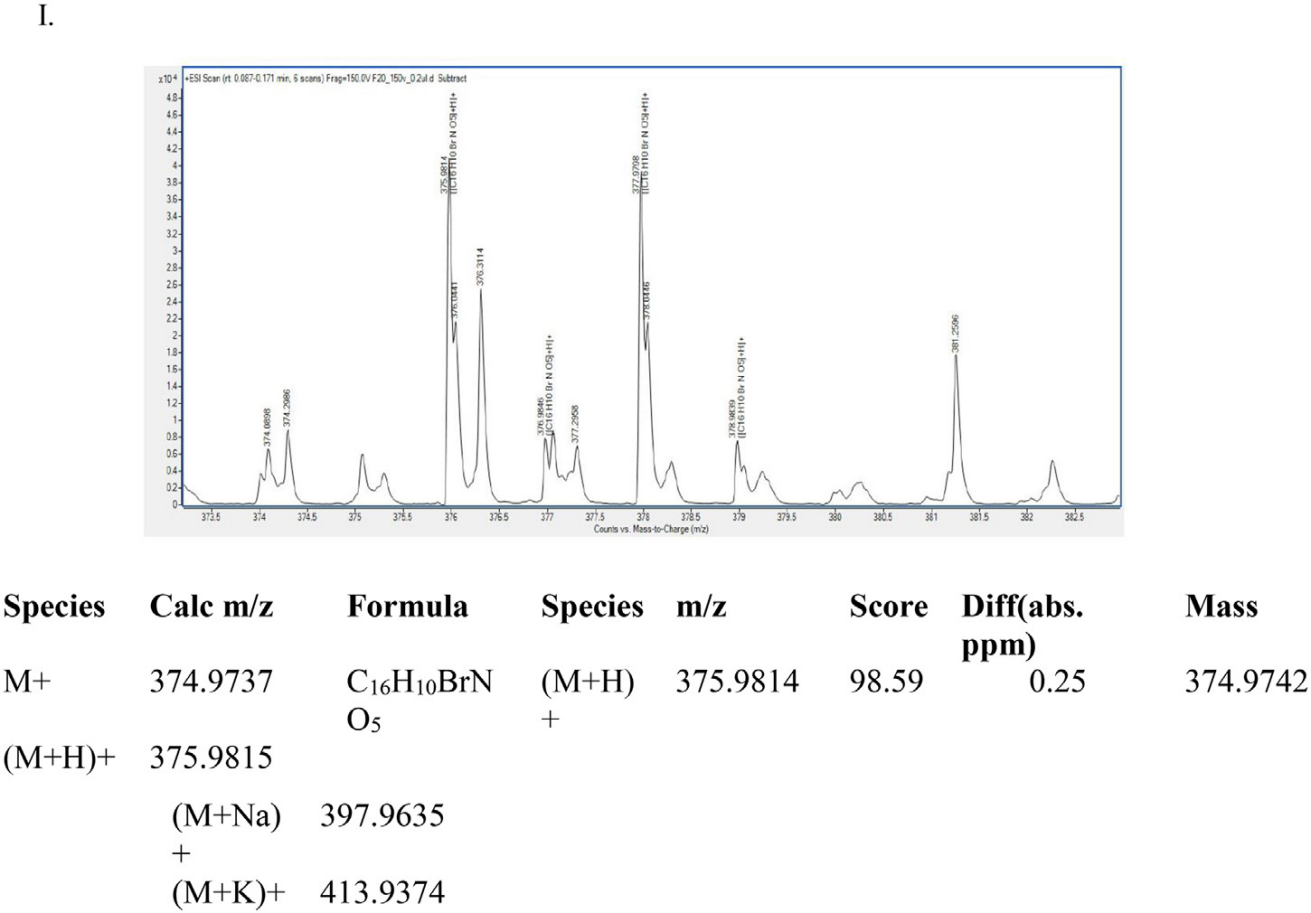
Proton (^1^H)-NMR and Carbon (^13^C)-NMR data for the three most active flavonols synthesized and characterized, namely, F9, F17 and F20. Data for all other flavonols are displayed in supplementary data 1. A. 3-Hydroxy-2-(3-methoxyphenyl)-7-methyl-4H-chromen-4-one (**F9**): ^1^H-NMR (400 MHz, CDCl_3_) B. 3-Hydroxy-2-(3-methoxyphenyl)-7-methyl-4H-chromen-4-one (**F9**): ^13^C-NMR (100 MHz; CDCl_3_) C. 3-Hydroxy-2-(3-methoxyphenyl)-7-methyl-4H-chromen-4-one (**F9**): HRMS (ESI) D. 2-(3-Chlorophenyl)-6-fluoro-3-hydroxy-4H-chromen-4-one (**F17**): ^1^H-NMR (400 MHz, CDCl_3_) E. 2-(3-Chlorophenyl)-6-fluoro-3-hydroxy-4H-chromen-4-one (**F17**): ^13^C-NMR (100 MHz; CDCl_3_) F. 2-(3-Chlorophenyl)-6-fluoro-3-hydroxy-4H-chromen-4-one (**F17**): HRMS (ESI) G. 2-(4-Bromophenyl)-3-hydroxy-6-methyl-8-nitro-4H-chromen-4-one (**F20**): ^1^H-NMR (400 MHz, CDCl_3_) H. 2-(4-Bromophenyl)-3-hydroxy-6-methyl-8-nitro-4H-chromen-4-one (**F20**): ^13^C-NMR (100 MHz; CDCl_3_) I. 2-(4-Bromophenyl)-3-hydroxy-6-methyl-8-nitro-4H-chromen-4-one (**F20**): HRMS (ESI)

**Fig. 2:**
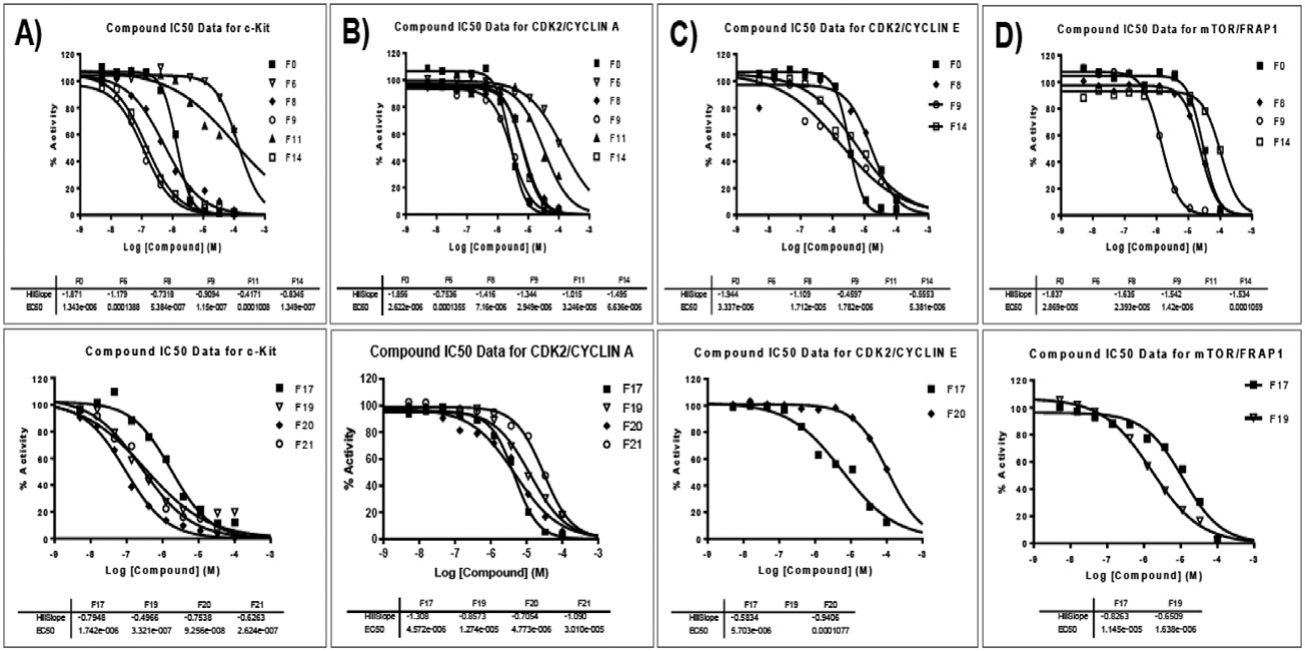
**Potent flavonol analogs significantly inhibit c-Kit, CDK2, and mTOR kinase activities.** Data from the determination of kinase inhibitory activity and IC_50_ values for potent flavonol analogs and fisetin. IC_50_ values (μM) calculated for potent inhibitors of: A) c-Kit, B) CDK2/Cyclin A, C) CDK2/Cyclin E and D) mTOR. Values are expressed as means ± SD of three independent assays, and all graphs were made using the GraphPad Prism program suite.

**Fig. 3:**
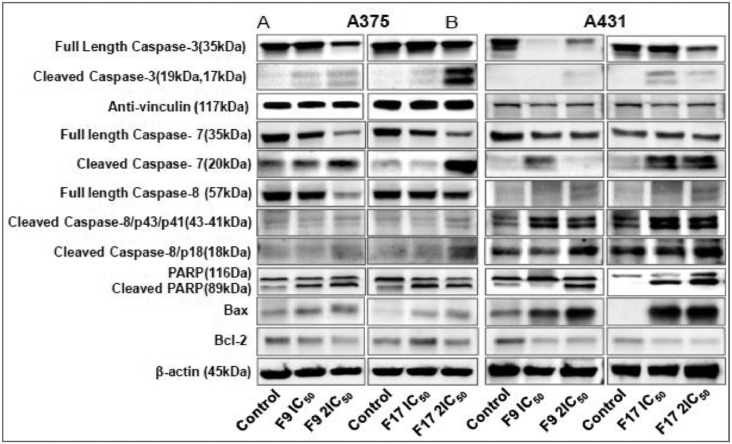

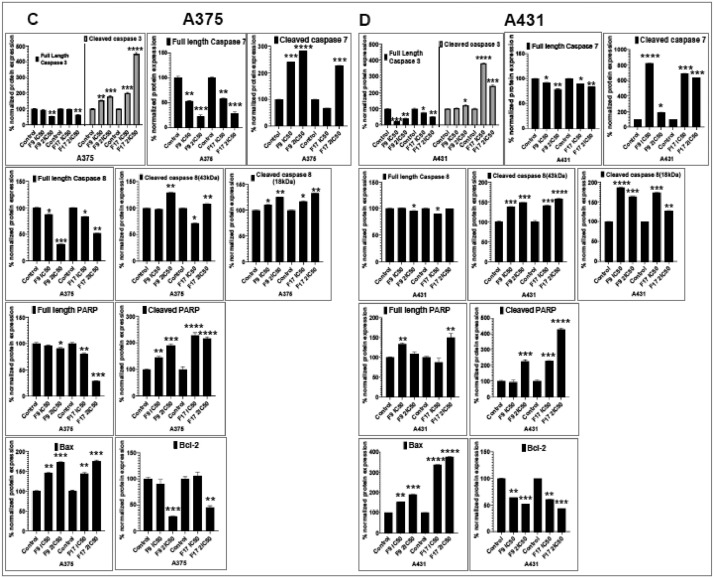
(A-B). Potent flavonol analogs induce apoptosis through activation of the extrinsic and intrinsic apoptotic pathways in melanoma and non-melanoma cells. Effect of different concentrations of flavonol analogs F9 and F17 on the protein expression levels of markers of apoptosis including pro- and cleaved caspase-3, -7 and -8, PARP (116 kDa) and cleaved PARP (85 kDa), as well as Bcl-2 family of proteins (Bax and Bcl-2), components of the intrinsic apoptosis pathway in 48h treated A375 and A431 cells. A375 and A431 cells were incubated in the absence or presence of flavonol analogs (F9 and F17; 0, IC_50_, 2xIC_50_; µM, 48h), and the whole-cell lysates of cells treated with/without F9 and F17 were subjected to SDS-polyacrylamide gel electrophoresis. Equal protein loading was confirmed by reprobing for β-actin or vinculin as loading control, and the actual protein levels were normalized to the loading control and expressed as percentage. The Western blot data shown are representative of immunoblot of more than two independent experiments with similar results. Fig. 3 (C-D). Potent flavonol analogs F9 and F17 induce apoptosis through activation of the extrinsic and intrinsic apoptotic pathways in melanoma and non-melanoma cells as shown graphically. The data expressed as the percentage of which analogs F9 and F17 significantly suppressed the protein expression levels of pro-and-cleaved caspases (3,7 and -8), PARP (116 kDa) and cleaved PARP (85 kDa), as well as Bax and Bcl-2 in (C) A375 and (D) A431 as compared with untreated control cells. Bar graphs represent mean ± SD of results of relative quantitative density values for the blots normalized with an internal loading control from three independent experiments. The statistical significance was determined using one-way ANOVA and Dunn's multiple comparison test, and *p < 0.05* (*) was considered significant.

**Fig. 4:**
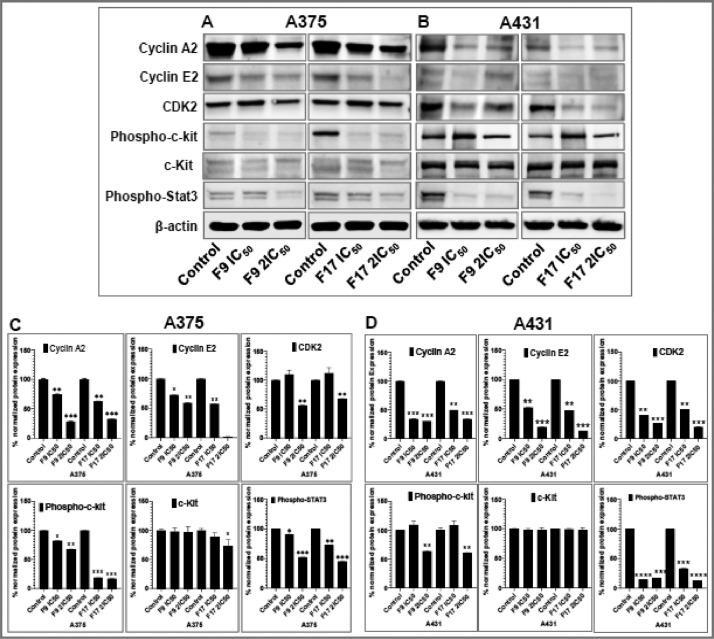
Potent flavonol analogs F9 and F17 inhibit the protein expression levels of cyclin A/E, CDK2 and phosphorylated c-Kit and Stat3 in A375 melanoma and A431 non-melanoma cells. Effect of different concentrations of flavonol analogs F9 and F17 on the protein expression levels of cyclin A2, cyclin E2, CDK2, phosphorylated c-Kit and Stat3 in 48h treated (A) A375 and (B) A431 cells. A375/A431 cells were incubated with/without analogs (F9 and F17; 0, IC_50_, 2xIC_50_; µM, 48h), and western blotting performed as described in the method section. Equal protein loading was confirmed by reprobing for β-actin as loading control, and protein levels were normalized to the loading control and expressed as percentage. The Western blot data shown are representative of immunoblots of three independent experiments with similar results. (C and D) The data expressed in the Bar graphs represent mean ± SD of relative quantitative normalized density values in percentage with an internal loading control from three independent experiments. The analogs F9 and F17 significantly suppressed the protein expression levels of these in (C) A375 and (D) A431 as compared with untreated control cells. For bar graphs, the statistical significance was determined using one-way ANOVA and Dunn's multiple comparison test, and *p < 0.05* (*) was considered significant.

**Fig. 5:**
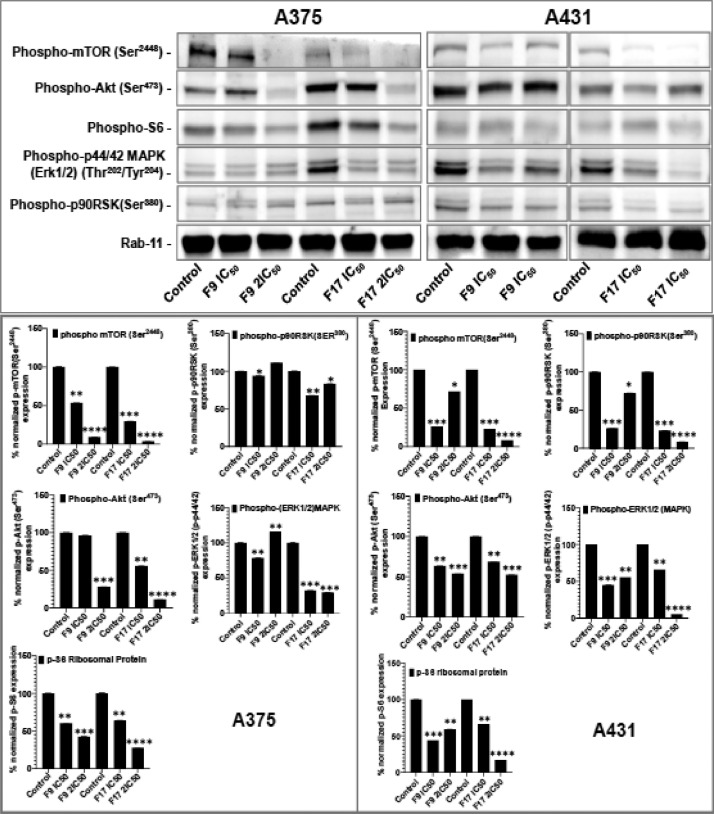
Potent flavonol analogs F9 and F17 inhibit the protein expression levels of phosphorylated p90RSK/Akt/mTOR/MAPK (ERK1/2) and ribosomal protein S6 in A375 melanoma and A431 non-melanoma cells. Role of the flavonol analogs F9 and F17 on the protein expression levels of cyclin A2, cyclin E2, CDK2, phosphorylated Akt, p90RSK, mTOR, MAPK (ERK1/2) and ribosomal protein in 48h treated (A) A375 and (B) A431 cells. A375/A431 cells were incubated with/without analogs (F9 and F17; 0, IC_50_, 2xIC_50_; µM, 48h), and western blotting performed as described in method section. Equal protein loading was confirmed by reprobing for β-actin as loading control, and protein levels were normalized to the loading control and expressed as percentage. The Western blot data shown are representative of immunoblot of three independent experiments with similar results (C and D). The data expressed in the Bar graphs represent mean ± SD of relative quantitative normalized density values in percentage with an internal loading control from three independent experiments. The analogs F9 and F17 significantly suppressed the protein expression levels of these in (C) A375 and (D) A431 as compared with untreated control cells. For bar graphs, the statistical significance was determined using one-way ANOVA and Dunn's multiple comparison test, and *p < 0.05* (*) was considered significant.

**Fig. 6:**
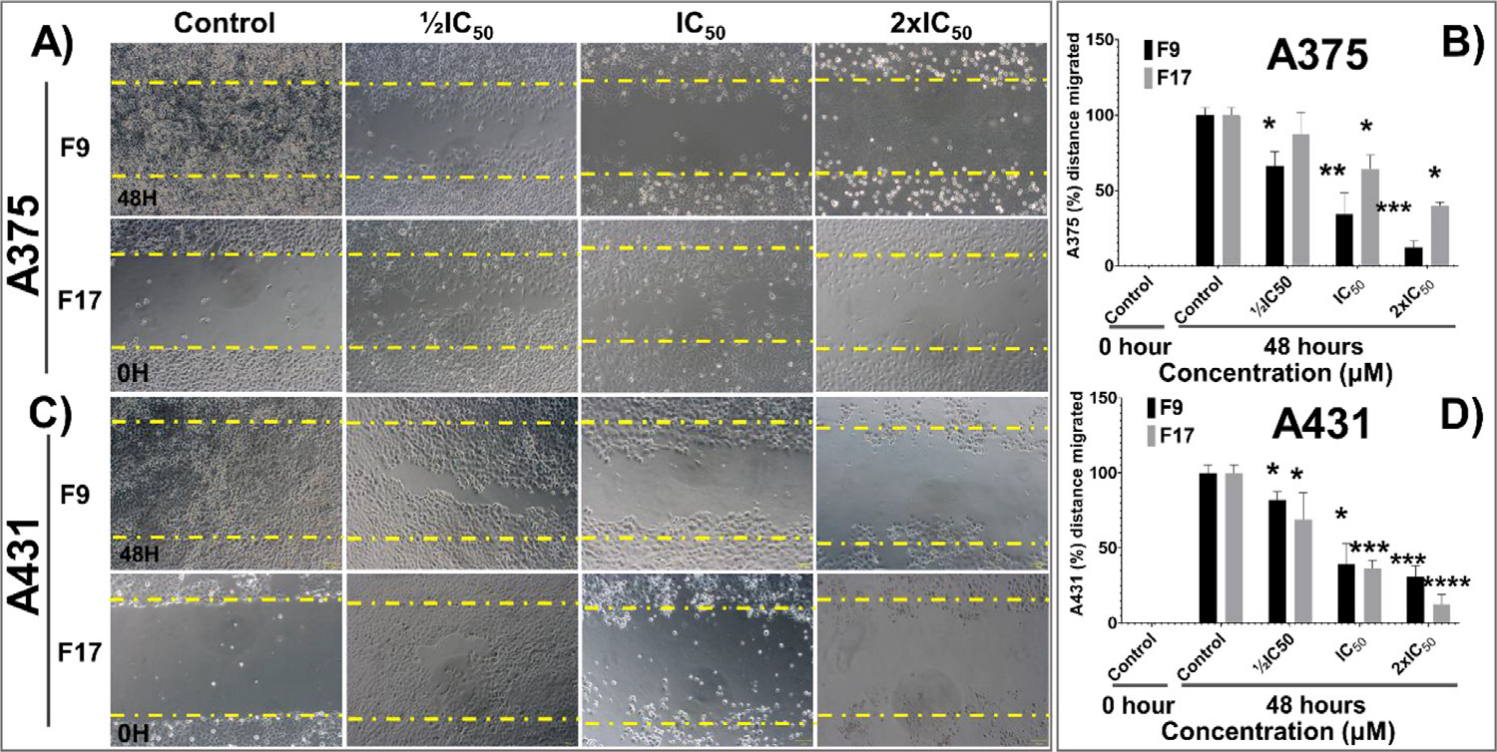
**Potent flavonol analogs F9 and F17 inhibit Scratch Wound Healing in 2D cultures of A375 and A431.** Effect of the flavonol analogs **F9** and **F17** on the wound closure into the initial cell-free areas compared to the percentage of cell-free areas **(**A) A375 and (B) A431 cells. (C and D) significant dose-dependent decrease in cultured cells’ scratch wound healing areas was observed in the presence of selected potent flavonol hits, compared to untreated control cells, after 48 h of incubation. The data expressed in the Bar graphs represent mean ± SD of scratched wound area values in percentage compared to 0h control and 24h controls from three independent experiments. The analogs **F9** and **F17** significantly suppressed wound healing area (C) A375 and (D) A431 as compared with untreated control cells. The statistical significance was determined using one-way ANOVA and Dunn's multiple comparison test, and *p < 0.05* (∗) was considered significant.

**Fig. 7:**
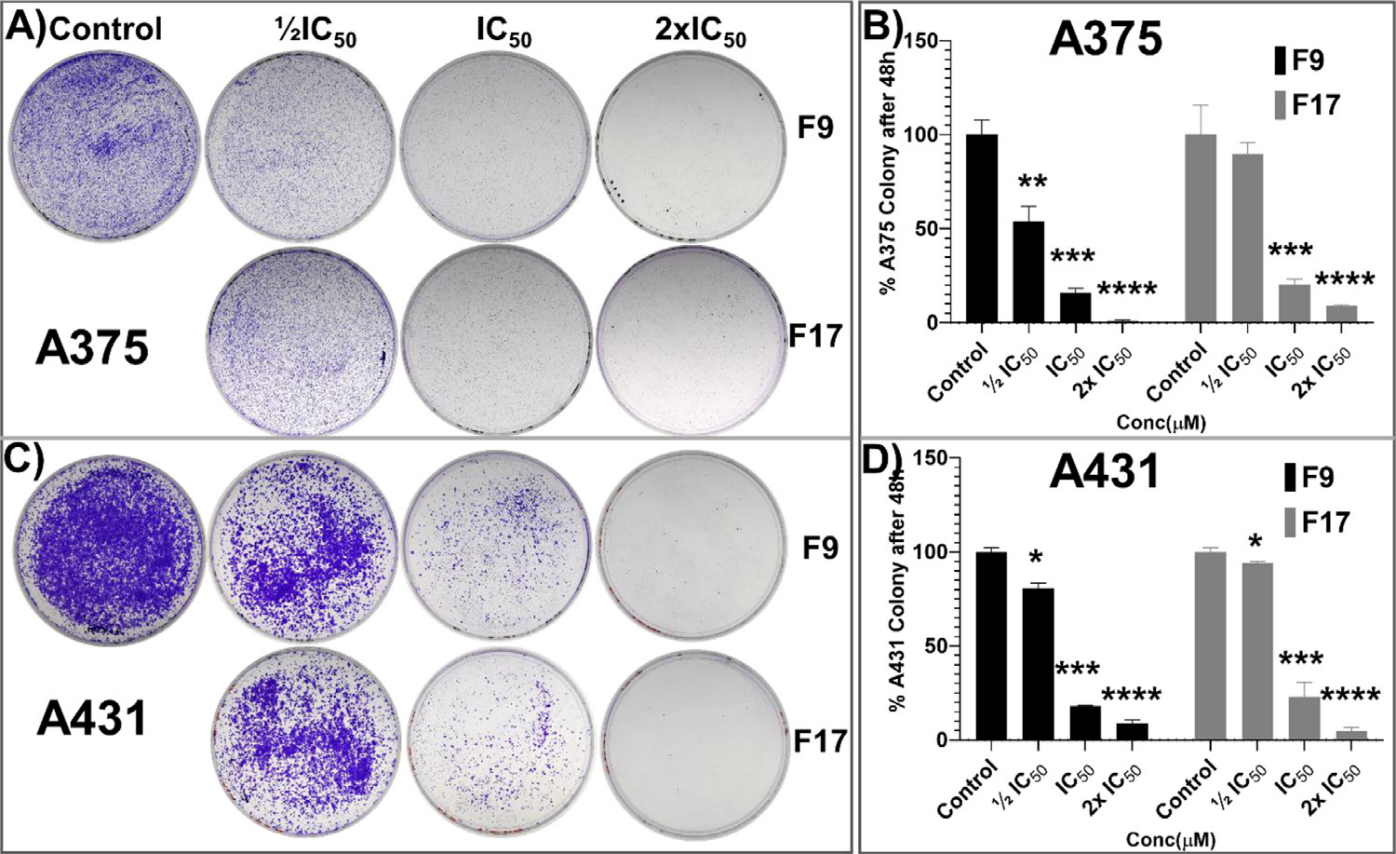
**Potent flavonol analogs F9 and F17 inhibit colony formation in 2D cultures of A375 and A431.** (A-D) Long-term effect of the potent flavonol analogs on clonogenic potential in (A) A375, and (B) A431 cells. Treatment with analogs **F9** and **F17** (½IC_50_, IC_50_ and 2xIC_50_ µM of the respective cell lines), significantly reduced/suppressed the percentage of colonies in a dose-dependent manner when compared to the respective control untreated cutaneous carcinoma cells. (C and D) The data expressed in the Bar graphs represent mean ± SD of values in percentage control from three-independent experiments, the statistical significance was determined using one-way ANOVA and Bonferoni's multiple comparison test, and *p < 0.05* (*) was considered significant.

This dataset article provides supplementary data to the associated research article by Roy et al., [1]. Fig. 1A-I reports the ^1^H-NMR, ^13^C-NMR, and HR-**MS** data for the three most active analogs **F9, F17** and **F20** [1]. The general approach for the synthesis of Fisetin analogs and their structures provided in Table 1. The full/detailed properties of all 24 flavonol analogs synthesized are provided in Supplementary dataset 1 and their raw NMR data files provided in ASCII file format and the raw HR-MS data files in .txt format linked in the (HR-MS Raw Files) in the source repository. Table 2 reports the results from the *in silico* prediction of absorption, distribution, metabolism, excretion (ADME) of the three potent fisetin analogs in the associated article (1). All 24 analogs were predicted to possess improved cell penetration properties, with the three most active being in the order as **F17**>**F9**>**F20**, compared to fisetin, the parent natural compound. The table also shows the lipophilicity profile for which all compounds have good oral bio-absorption and intestinal absorption, but with less effective sub-lingual absorption. The Table 2 dataset presents on the three most active analogs, based on the log Kp value, is favored as follows **F17**>**F9**>**F20** respectively, as compared to fisetin (**F0**). This reveals an important property for the skin pharmaceutical applications, an indication that each derivative has a better skin permeation (cm/s) ability than the parent compound, fisetin. Detailed raw ADME prediction data for all 24 flavonol compounds synthesized are displayed in supplementary dataset 2. Tables 3–14 report results of RMSD values obtained after Cross-docking/re-docking for the selection of PDB for EGFR (Table 2), c-Kit (Table 4), Akt (Table 5), MET (Table 6), MEK1 (Table 7), VEGFR1 (Table 8), MAPK (Table 9), mTOR (Table 10), PI3K (Table 11), PIP5K1α (Table 12), FGFR (Table 13), and CDK2 (Table 14) related to the parent article(1). Fig. 2 reports potency data analyzed from examination of the inhibitory activities of the selected, most active analogs compared to fisetin on the following kinases: A) c-Kit, B) CDK2/Cyclin A, C) CDK2/Cyclin E and D) mTOR. The attributes of IC_50_ values (μM) of calculated ranked inhibitor potency values are expressed as means ± SD of three independent assays and are listed and described in Table 3 of the related research article [1]. Fig. 3 reports the mean western blot bands (A-B) and their corresponding densitometric data analysis in bar graph format (C-D) of the different melanoma (A375) and non-melanoma (A431) cells treated in the presence or absence of test analogs **F9** and **F17**, showing the differential expression of apoptosis induction via activation of the extrinsic and intrinsic apoptotic pathways. The data for **F20** are described in the related research article [1]. Fig. 4 summarizes the Western blot band results (A-B) and the Bar graph plots data (C-D) from the analysis of the effects of different concentrations of flavonol analogs **F9** and **F17** on the protein expression levels of cyclin A2, cyclin E2, CDK2, phosphorylated c-Kit and Stat3 in 48h treated A375 and A431 skin cancer cells. In Fig. 5, the Western blot bands (Upper panel) and the Bar graph plots (lower panels) exhibit analyzed data from the effects of different concentrations of analogs **F9** and **F17** on the protein expression levels of phosphorylated p90RSK/Akt/mTOR/MAPK (ERK1/2) and ribosomal protein S6 in A375 melanoma and A431 non-melanoma skin cancer cells after 48h treatment. The supplementary raw data are available in in the link. Fig. 6 reports the micrographic photo images (A-B) and the mean values (C-D) from the analysis of the dose-dependent effects of the analogs **F9** and **F17** on the wound closure into the initial cell-free areas compared to the percentage of the cell-free areas (A) A375 and (B) A431, in contrast to untreated control cells, after 48 h of incubation (see supplementary raw data files are available at the link). Fig. 7 describes the data from the determination of the long-term dose-dependent effect of the analogs **F9** and **F17** on the percentage of colonies when compared to the respective untreated cutaneous carcinoma control cell lines. All the data for compound **F20** are displayed in the associated/parent research article [1]. The supplementary raw data files are available in the link.

**Table 1:** Synthesis of Fisetin analogs *via* microwave-assisted Claisen-Schmidt condensation and modified Algar-Flynn-Oyamada (AFO) reactions.

**Table 2:** *In silico* prediction of absorption, distribution, metabolism and excretion (ADME) for the most potent fisetin analogs, namely **F9, F17** and **F20**.

Properties	Cmpd#	1	10	18	21
	**Code**	F0	**F9**	**F17**	**F20**
	**MW**	286.24	282.29	290.67	376.16
**Physicochemical**	**#Heavy atoms**	21	21	20	23
	**#Aromatic heavy atoms**	16	16	16	16
	**Fraction Csp3**	0	0.12	0	0.06
	**#Rotatable bonds**	1	2	1	2
	**#H-bond acceptors**	6	4	4	5
	**#H-bond donors**	4	1	1	1
	**MR**	76.01	81.4	74.91	91.43
	**TPSA**	111.13	59.67	50.44	96.26
**Lipophilicity**	**iLOGP**	1.5	2.98	2.74	2.46
	**XLOGP3**	1.97	3.37	3.76	3.92
	**WLOGP**	2.28	3.48	4.38	4.14
	**MLOGP**	−0.03	1.57	2.57	1.53
	**Silicos-IT Log P**	2.03	4.03	4.54	2.48
	**Consensus Log P**	1.55	3.09	3.6	2.91
**Water solubility**	**ESOL Log S**	−3.35	−4.15	−4.54	−5.02
	**ESOL Solubility (mg/ml)**	1.27E-01	2.02E-02	8.44E-03	3.55E-03
	**ESOL Solubility (mol/l)**	4.43E-04	7.16E-05	2.90E-05	9.45E-06
	**ESOL Class**	Soluble	Moderately soluble	Moderately soluble	Moderately soluble
	**Ali Log S**	−3.93	−4.3	−4.51	−5.64
	**Ali Solubility (mg/ml)**	3.37E-02	1.41E-02	8.94E-03	8.61E-04
	**Ali Solubility (mol/l)**	1.18E-04	5.00E-05	3.07E-05	2.29E-06
	**Ali Class**	Soluble	Moderately soluble	Moderately soluble	Moderately soluble
	**Silicos-IT LogSw**	−3.82	−6.07	−6.44	−6.11
	**Silicos-IT Solubility (mg/ml)**	4.29E-02	2.43E-04	1.05E-04	2.89E-04
	**Silicos-IT Solubility (mol/l)**	1.50E-04	8.61E-07	3.61E-07	7.69E-07
	**Silicos-IT class**	Soluble	Poorly soluble	Poorly soluble	Poorly soluble
**Pharmaceutics**	**GI absorption**	High	High	High	High
	**BBB permeant**	No	Yes	Yes	No
	**Pgp substrate**	No	No	No	No
	**CYP1A2 inhibitor**	Yes	Yes	Yes	Yes
	**CYP2C19 inhibitor**	No	Yes	Yes	No
	**CYP2C9 inhibitor**	No	Yes	No	Yes
	**CYP2D6 inhibitor**	Yes	Yes	No	No
	**CYP3A4 inhibitor**	Yes	Yes	Yes	Yes
	**log Kp (cm/s) (Skin permeation)**	−6.65	−5.63	−5.4	−5.81
**Drug-likeness**	**Lipinski #violations**	0	0	0	0
	**Ghose #violations**	0	0	0	0
	**Veber #violations**	0	0	0	0
	**Egan #violations**	0	0	0	0
	**Muegge #violations**	0	0	0	0
	**Bioavailability Score**	0.55	0.55	0.55	0.55
	**PAINS #alerts**	1	0	0	0
	**Brenk #alerts**	1	0	0	2
	**Leadlikeness #violations**	0	0	1	2
	**Synthetic Accessibility**	3.16	3.19	2.84	3.29

**Table 3:** RMSD values obtained after Cross-docking for the selection of PDB for EGFR.

PDB ID						
Ligand	4LQM	3W2S	1M17	3W2R	4I21	5XDL
4LQM	1.35	2.54	1.03	6.53	3.69	1.67
3W2S	5.85	1.65	2.05	6.18	4.82	1.35
1M17	1.37	2.42	0.49	2.47	3.31	2.59
3W2R	6.33	1.83	1.23	1.34	2.09	2.87
4I21	1.94	3.41	2.08	2.96	0.93	3.56
5XDL	1.25	4.36	1.69	3.51	1.87	2.41
Average	3.01	2.70	1.42	3.83	2.78	2.00

*average RMSD value showed best fitting for PDB ID: 1M17, which is selected for further study.

**Table 4:** RMSD values obtained after Cross-docking for the selection of PDB for c-Kit.

PDB ID						
Ligand	6GQM	6KLA	6XV9	6GQJ	6GQK	6GQL
6GQM	0.77	1.22	1.89	1.08	3.85	2.65
6KLA	1.02	2.61	2.71	1.25	2.29	1.48
6XV9	1.90	1.79	0.93	2.06	1.14	3.72
6GQJ	2.52	2.64	4.60	0.82	1.67	3.75
6GQK	3.03	2.29	2.27	1.52	1.10	2.77
6GQL	4.35	3.00	1.17	2.83	2.07	1.96
Average	2.26	2.25	2.26	1.54	2.02	2.72

* average RMSD value showed best fitting for PDB ID: 6GQJ, which is selected for further study.

**Table 5:** RMSD values obtained after Cross-docking for the selection of PDB for Akt.

PDB ID						
Ligand	3D0E	1H10	1UNQ	3O96	2 × 39	2XH5
3D0E	1.72	2.80	4.07	5.78	3.96	3.45
1H10	5.58	1.54	3.98	6.18	5.14	5.89
1UNQ	3.95	4.57	1.49	4.27	4.83	3.26
3O96	5.36	3.48	2.42	3.40	2.68	2.92
2 × 39	4.79	4.12	3.16	4.98	0.93	3.62
2XH5	3.85	5.45	3.76	5.46	2.28	1.38
Average	4.20	3.66	3.14	5.01	3.30	3.42

* average RMSD value showed best fitting for PDB ID:1UNQ, which is selected for further study.

**Table 6:** RMSD values obtained after Cross-docking for the selection of PDB for MET.

PDB ID											
Ligand	2WGJ	3CCN	3CD8	3DKF	3DKG	3F66	3I5N	3QTI	3U6H	5EYD	1R0P
2WGJ	0.77	8.2	0.89	1.08	1.51	−	0.84	2.42	11.37	0.84	9.09
3CCN	1.02	0.61	5.71	0.52	0.95	−	1.37	0.47	6.91	1.59	3.11
3CD8	0.90	5.79	1.83	0.68	2.31	7.27	0.62	0.79	6.71	0.63	5.99
3DKF	7.5	7.64	5.60	4.82	5.76	5.37	7.78	6.64	8.13	5.70	9.66
3DKG	4.03	5.29	7.27	5.25	−	2.67	8.74	7.21	−	6.88	9.11
3F66	10.35	9.00	7.71	3.98	6.07	−	7.67	8.25	12.07	7.66	11.49
3I5N	2.95	2.39	0.83	0.88	1.81	−	0.60	5.66	7.29	3.88	5.89
3QTI	−	−	7.06	−	7.16	−	−	−	7.99	10.72	−
3U6H	10.49	−	11.48	10.7	10.99	−	11.86	13.27	1.92	15.19	15.91
5EYD	0.89	0.83	0.96	0.94	1.40	−	0.96	0.86	7.82	0.61	7.35
1R0P	5.70	8.26	3.47	4.49	4.07	−	5.84	5.85	6.01	5.90	0.26
Average	4.46	4.51	4.61	3.33	4.20	−	4.62	5.12	7.62	5.42	7.78

*average RMSD value showed best fitting for PDB ID:3DKF, which is selected for further study.

**Table 7:** RMSD values obtained after Cross-docking for the selection of PDB for MEK1.

PDB ID					
Ligand	3EQC	4LMN	3DV3	5EYM	4U81
3EQC	1.13	2.54	2.03	2.52	3.94
4LMN	1.55	1.21	1.46	1.86	4.18
3DV3	2.43	1.74	0.90	2.64	3.31
5EYM	3.26	3.68	1.42	1.44	2.28
4U81	1.88	1.92	2.86	3.32	0.93
Average	2.05	2.21	1.73	2.35	2.92

* average RMSD value showed best fitting for PDB ID:3DV3, which is selected for further study.

**Table 8:** RMSD value obtained after re-docking for the selection of PDB for VEGFR1.

PDB ID	
Ligand	3HNG
3HNG	0.82

**Table 9:** RMSD values obtained after Cross-docking for the selection of PDB for MAPK.

PDB ID								
Ligand	1PME	6OPH	3SA0	6OPI	4ZXT	3W55	4QP2	5BUJ
1PME	1.05	2.45	2.03	1.86	3.68	2.77	3.99	2.94
6OPH	1.58	1.20	1.58	1.98	2.89	4.31	2.83	2.51
3SA0	1.95	2.44	0.89	2.22	3.18	3.62	2.81	1.81
6OPI	2.54	1.69	1.44	3.04	1.77	2.81	1.73	1.96
4ZXT	4.79	2.36	1.68	3.58	0.93	1.99	2.51	2.79
3W55	3.21	4.65	1.99	1.87	2.34	1.83	3.23	3.66
4QP2	2.68	2.18	1.37	2.65	1.73	2.63	1.51	3.75
5BUJ	3.18	2.66	3.12	3.69	2.47	2.39	1.97	1.35
Average	2.62	2.45	1.76	2.61	2.37	2.79	2.57	2.59

* average RMSD value showed best fitting for PDB ID:3SA0, which is selected for further study.

**Table 10:** RMSD value obtained after re-docking for the selection of PDB for mTOR.

PDB ID	
Ligand	2NPU
2NPU	4.82

**Table 11:** RMSD values obtained after Cross-docking for the selection of PDB for PI3K.

PDB ID					
Ligand	4FAD	4FA6	4TV3	4TUU	6OAC
4FAD	1.16	2.32	2.07	4.67	3.19
4FA6	1.85	1.44	2.02	3.06	4.14
4TV3	2.53	2.08	0.59	3.11	3.31
4TUU	2.49	1.91	1.62	1.36	2.75
6OAC	4.79	3.34	1.38	2.49	0.93
Average	2.56	2.21	1.53	2.93	2.86

* average RMSD value showed best fitting for PDB ID:4TV3, which is selected for further study.

**Table 12:** RMSD values obtained after Cross-docking for the selection of PDB for PIP5K1α.

PDB ID							
Ligand	6CN3	6CN2	6CMW	5E3T	4TZ7	5E3S	5E3U
6CN3	0.87	2.80	2.59	1.08	2.11	3.37	1.48
6CN2	1.32	1.70	1.57	0.72	2.99	2.23	1.73
6CMW	1.89	3.97	1.75	0.88	2.21	1.27	2.62
5E3T	1.75	2.64	2.65	0.62	1.36	1.68	3.78
4TZ7	2.03	3.29	1.27	2.12	1.15	1.24	2.17
5E3S	3.51	2.90	1.17	3.98	3.03	1.38	2.27
5E3U	2.59	1.93	3.83	0.89	1.61	2.42	1.70
Average	1.99	2.74	2.11	1.47	2.06	1.94	2.25

*average RMSD value showed best fitting for PDB ID:5E3T, which is selected for further study.

**Table 13:** RMSD values obtained after Cross-docking for the selection of PDB for FGFR.

PDB ID						
Ligand	4UWC	5AM6	5EW8	5AM7	4UXQ	4WUN
4UWC	1.37	2.33	2.91	3.73	1.69	2.76
5AM6	1.85	2.01	2.41	1.86	3.26	1.87
5EW8	1.95	3.11	1.39	2.71	3.81	3.36
5AM7	2.19	1.81	2.57	1.63	1.89	2.43
4UXQ	3.94	2.61	1.61	1.87	0.93	1.97
4WUN	2.83	2.37	2.26	2.93	4.57	1.55
Average	2.35	2.37	2.19	2.45	2.69	2.32

* average RMSD value showed best fitting for PDB ID:5EW8, which is selected for further study.

**Table 14:** RMSD values obtained after Cross-docking for the selection of PDB for CDK2.

PDB ID											
Ligand	1B39	1GIJ	1GII	2DUV	1GIH	2C5Y	1B38	3PXY	6INL	4EZ3	6GUH
1B39	1.22	2.81	1.89	1.08	1.51	1.44	0.84	2.42	5.37	0.84	5.09
1GIJ	2.01	1.16	1.57	0.52	0.95	1.87	1.37	0.47	4.91	1.59	1.21
1GII	1.92	1.79	3.18	0.68	2.31	5.27	2.62	0.79	3.71	0.63	3.99
2DUV	2.25	3.64	2.46	2.82	3.76	3.37	3.78	4.64	5.13	3.70	5.43
1GIH	3.04	−	2.72	3.25	−	2.67	6.74	5.21	−	4.88	4.21
2C5Y	3.51	−	2.17	1.98	4.07	−	3.67	6.25	6.07	4.66	4.49
1B38	−	3.92	1.83	0.88	1.81	3.59	2.60	3.66	3.29	3.88	3.89
3PXY	2.59	3.29	2.06	−	5.16	1.66	−	−	4.99	4.72	−
6INL	4.19	3.11	5.48	4.72	4.99	3.96	4.86	7.27	1.92	1.19	3.72
4EZ3	0.89	1.38	1.06	0.94	1.40	−	4.96	0.86	2.82	0.61	3.13
6GUH	2.50	3.62	3.47	2.49	2.07	2.64	3.84	3.85	3.01	3.90	0.46
Average	2.41	2.74	2.53	1.9	2.8	2.94	3.60	3.54	4.12	2.78	3.56

* average RMSD value showed best fitting for PDB ID:2DUV, which is selected for further study.

## 2 Experimental Design, Materials and Methods

This study's major purpose was to validate the newly synthesized and fully characterized flavonol analogs [4], as anticancer agents, with different effects on basal and squamous carcinoma of the skin cells *in vitro*. Inclusion criteria included in the supplementary datasets were not extensively presented in the associated article. These data complement and further enhance the results presented in the associated article [1]. These datasets and their analyses aimed at improving our understanding of the physicochemical and other pharmaceutical properties of the compounds *in vitro* were explored through the use of the online SWISS-ADME platform of the Swiss Institute of Bioinformatics (http://www.swissadme.ch) [3], and compared with the same descriptors obtained for fisetin, the reference compound. A detailed description of the procedures of study-specific investigation and the molecular and cellular assay protocols are essentially as presented in the related research article [1]. Equal protein loading was confirmed by reprobing for β-actin or vinculin as a loading control, and the actual protein levels were normalized to the loading control and expressed as a percentage. The Western blot data shown are representative of immunoblot of more than two independent experiments with similar results. Statistical analysis was conducted, and graphics were designed using GraphPad PRISM program suite, version 8 (GraphPad Software, Inc., La Jolla, CA, USA). The data expressed in the Bar graphs represent mean ± SD of values in percentage control from three independent experiments; the statistical significance was determined using one-way ANOVA and Bonferoni's multiple comparison test or Dunn's multiple comparison test. P-values ≤ 0.05 were considered statistically significant.

## Ethics Statement

The primary normal human epidermal keratinocytes used were isolated from new born foreskin biopsies or adult skin biopsies and established in culture using published protocols, [5], and were obtained under a University of Wisconsin–Madison-approved institutional review board protocol. The experiments were conducted following the Declaration of Helsinki principles.

## CRediT Author Statement

**Tithi Roy:** Methodology, Investigation, Writing – Original draft Preparation, Writing – review & editing; **Samuel T. Boateng:** Methodology, Investigation, Writing – review & editing; **Sergette Banang-Mbeumi:** Methodology, Investigation, Writing – review & editing; **Pankaj K. Singh:** Methodology, Investigation, Formal analysis, Writing – review & editing; **Pratik Basnet:** Methodology, Investigation, Formal analysis, Writing – review; **Roxane-Cherille N. Chamcheu:** Methodology, Investigation, Writing – review & editing **Federico Ladu:** Methodology, Investigation, Writing & review; **Isabel Chauvin:** Methodology and Investigation; **Vladimir S. Spiegelman:** Resources, Formal analysis, Writing – review & editing; **Ronald A. Hill:** Investigation, Formal analysis, Writing – review & editing; **Konstantin G. Kousoulas:** Investigation, supervision, Resources, Writing – review & editing.**Bolni Marius Nagalo:** Methodology, Investigation, supervision, Writing – review & editing; **Anthony L. Walker:** Methodology, Investigation, supervision, Writing – review & editing; **Jean Fotie:** Methodology, Investigation, Writing – Original draft Preparation, Writing – review & editing; **Siva Murru:** Methodology, Investigation, supervision, Resources, Writing – review & editing; **Mario Sechi:** Methodology, Investigation, supervision, Resources, Writing – Original draft Preparation, Writing – review & editing; **Jean Christopher Chamcheu:** Conceptualization, Methodology, Investigation, Resources, Formal analysis, Writing – Original draft Preparation, Writing – review & editing, supervision, Visualization.

## Declaration of Competing Interest

The authors declare that they have no known competing financial interests or personal relationships which have or could be perceived to have influenced the work reported in this article.

## Appendix Supplementary materials

Image, application 1

Image, application 2
